# Spatial heterogeneity lowers rather than increases host–parasite specialization

**DOI:** 10.1111/jeb.12689

**Published:** 2015-07-22

**Authors:** E. Hesse, A. Best, M. Boots, A. R. Hall, A. Buckling

**Affiliations:** ^1^ ESIBiosciencesUniversity of ExeterPenrynUK; ^2^ School of Mathematics and StatisticsUniversity of SheffieldSheffieldUK; ^3^ CLESBiosciencesUniversity of ExeterPenrynUK; ^4^ ETH ZürichZürichSwitzerland

**Keywords:** antagonistic coevolution, bacteria, migration, phages, selection mosaics, specificity

## Abstract

Abiotic environmental heterogeneity can promote the evolution of diverse resource specialists, which in turn may increase the degree of host–parasite specialization. We coevolved *Pseudomonas fluorescens* and lytic phage *ϕ*2 in spatially structured populations, each consisting of two interconnected subpopulations evolving in the same or different nutrient media (homogeneous and heterogeneous environments, respectively). Counter to the normal expectation, host–parasite specialization was significantly lower in heterogeneous compared with homogeneous environments. This result could not be explained by dispersal homogenizing populations, as this would have resulted in the heterogeneous treatments having levels of specialization equal to or greater than that of the homogeneous environments. We argue that selection for costly generalists is greatest when the coevolving species are exposed to diverse environmental conditions and that this can provide an explanation for our results. A simple coevolutionary model of this process suggests that this can be a general mechanism by which environmental heterogeneity can reduce rather than increase host–parasite specialization.

## Introduction

Both theoretical and experimental studies show that migration across spatially heterogeneous environments can promote the evolution of diverse specialists within single populations as a result of divergent selection and local adaptation, and subsequent mixing (Hedrick, 1986; Futuyma & Moreno, 1988; Kassen, 2002; Lenormand, 2002; Venail *et al*., 2008). Intuitively, such within‐population diversity of specialists resulting from spatial heterogeneity could also lead to the evolution of parasites (or other exploiters) specializing on different hosts. Surprisingly, this idea has not been directly investigated. Theoretical work has shown that spatial heterogeneity in the physical environment can influence local adaption in coevolving systems (Gomulkiewicz *et al*., 2000; Thompson, 2005; Gandon & Nuismer, 2009). Moreover, data from a range of natural systems (Thompson, 2005; Laine, 2006; Toju & Sota, 2006; Thrall *et al*., 2007; Vale *et al*., 2008; Koskella *et al*., 2011; Laine *et al*., 2014) and experimental evolution studies using rapidly coevolving bacteria and viruses (Forde *et al*., 2004, 2007; Benmayor *et al*., 2009; Vogwill *et al*., 2009; Lopez‐Pascua *et al*., 2010, 2012; Sieber *et al*., 2013) are consistent with a key role of spatial heterogeneity in the evolution of host–parasite specialization. However, whether spatially heterogeneous environments increase within‐population specialization in host–parasite interactions has yet to be addressed.

Here, we tested this hypothesis by coevolving *Pseudomonas fluorescens* and lytic phage *ϕ*2 (Buckling & Rainey, 2002) in spatially structured populations consisting of two interconnected subpopulations evolving in the same or different nutrient media. Previous work on this system has provided a direct demonstration of long‐term antagonistic coevolution in the absence (Buckling & Rainey, 2002) and the presence of migration (e.g. Morgan *et al*., 2005; Lopez‐Pascua *et al*., 2010). To maximize possible effects of environmental heterogeneity on the evolution of within‐population specialization, we chose environments (different types of media) between which we previously observed strong specialization of parasites on their hosts, as evidenced by parasite local adaptation (phages performing better on their own hosts than hosts evolved in the alternative environment) (Lopez‐Pascua *et al*., 2012). We predicted that migration between these environments is likely to increase the amount of host–parasite specialization within each environment. The media types used were 0.5× and 5× the normal concentration of a standard microbiological medium, Kings Media B, typically used to culture Pseudomonads (King *et al*., 1954). Peak densities are achieved at 1× normal concentration, and a toxic effect of 5× concentration results in comparable densities, mean levels of infectivity and resistance evolution as for 0.5× medium.

**Figure 1:**
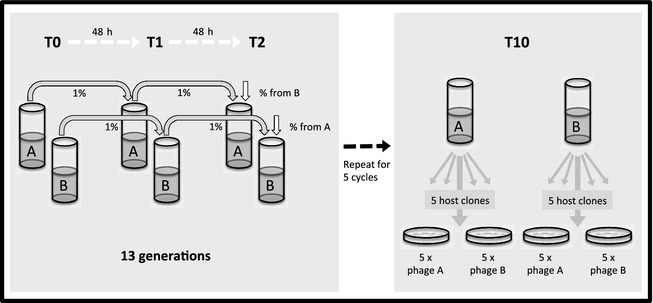
Design of the coevolution experiment testing the effects of heterogeneity and migration on the evolution of host–parasite specialization. In short, two paired communities (A and B) coevolved in parallel in KB broth containing the same or different concentrations of glycerol and peptone (0.5× and 5× the normal concentration). Every second transfer (T2), migration was carried out by reciprocally transferring 1% of the community between the paired environments. For the heterogeneous environment, we also imposed a high migration rate (50%), yielding four selection regimes in total. After ten transfers (T10), resistance and infectivity interactions were measured between multiple bacteria and phage clones within each community.

We started by independently coevolving communities in their respective environments for approximately 13 generations, during which time extensive coevolution occurs (Buckling & Rainey, 2002). Migration was then imposed every 13 generations by transferring 1% or 50% of the total community between paired environments (Fig. 1). This procedure was repeated for five cycles (approximately 65 generations), after which resistance and infectivity interactions were measured between multiple bacteria and phage clones within each experimental population. Note that the effect of differential migration rates on host–parasite specialization was only tested for the heterogeneous communities, yielding four different selection regimes in total. Contrary to the general expectation, we found that environmental heterogeneity resulted in the evolution of lower host–parasite specialization, that is a narrow range of different phenotypes each specialized to infect/resist different hosts/parasites. This result could not simply be explained by dispersal homogenizing populations, as this would have resulted in the heterogeneous treatments having levels of specialization equal to or greater than that of the homogeneous environment with the lowest level of specialization**.** We developed a simple coevolutionary model that shows that these results can be explained by increased selection for generalism in abiotic heterogeneous environments.

## Materials and methods

### Selection experiment

Bacteria and phages were cultured in 25 mL glass universals containing 6 mL of M9 salt solution with two different concentrations of glycerol and proteose peptone (0.5 and 5 times the standard concentration for King's Media B (KB) of 10 g L^−1^ glycerol and 20 g L^−1^ proteose peptone) (Lopez‐Pascua & Buckling, 2008). Propagation in these different media results in divergence in resistance and infectivity traits, such that phages are much better at infecting bacteria grown in the same media than different media (Lopez‐Pascua *et al*., 2012). We employed four different selection regimes: 1% reciprocal migration between paired subpopulations evolving in (a) 0.5× M9 salts, (b) 5× M9 salts, (c) 0.5× and 5× M9 salts and (d) 50% migration between 0.5× and 5× M9 salts media (Fig. 1). For each treatment, 12 structured replicate populations were established by inoculating two paired microcosms with 10^8^ bacterial cells (derived from a single *P. fluorescens* SBW25 clone grown overnight in KB at 28 °C, in an orbital shaker at 200 rpm), and 10^5^ phage particles, obtained from a single plaque of a clonal phage *ϕ*2. Bacteria and phages were then allowed to coevolve at 28 °C unshaken for 48 h before 1% (60 μL) of the total population was transferred into fresh microcosms, following vortex mixing to homogenize the culture. Every second transfer, we additionally imposed 1% or 50% reciprocal migration, meaning that populations coevolved independently for approximately 13 generations before being diluted into fresh microcosms together with the paired subpopulation. At this time, we also froze samples of the total population at −80 °C in 20% (v:v) glycerol solution and isolated phages by centrifuging cultures with 10% chloroform, which lyses and pellets bacterial debris (Buckling & Rainey, 2002). The process was repeated for a total of 10 transfers (approximately 65 bacterial generations).

For each structured population, we measured the resistance of 10 bacterial clones against infection by 10 phage clones (i.e. five bacteria and phage clones from each subpopulation; Fig. 1). Bacterial clones were isolated by plating diluted culture onto KB agar, whereas phage clones were isolated by plating onto a 1% bacterial lawn grown on a KB soft agar plate containing the appropriate M9 salts concentration (Scanlan *et al*., 2011). Resistance was determined by spotting 5 μL of isolated phage onto a single colony bacterial lawn. A colony was defined as resistant if there was no inhibition of growth. Mean phage infectivity of a given individual subpopulation, or population as a whole, was calculated as the average of the number of host clones that each isolated phage clone can infect. These assays were performed after 10 transfers.

### Data analyses

To test how dispersal across an environmental gradient affects host–parasite specificity, we partitioned total within‐population variance of individual performances into *V *= *V*_P_ *+ V*_B_ + *V*_PB_, where *V*_P_ is variation in mean individual infectivity among phage clones, *V*_B_ is variation in phage infectivity across different bacterial clones, and *V*_PB_ is the phage–bacterium clone‐by‐clone interaction. This latter term arises if phage clones react differently to different bacterial clones and is a measure of the amount of niche variation within populations. *V*_PB_ variance can be further broken down into responsiveness (*R*) and inconsistency (*I*), which are relative measures of the diversity of resource exploitation strategies and niche differentiation, respectively (e.g. Bell, 1990; Barrett *et al*., 2005; Hall & Colegrave, 2007; Venail *et al*., 2008). Inconsistency was calculated for all pair‐wise phage–bacterium clone combinations: *σ*_G1_ *σ*_G2_ × (1−*ρ*_G1G2_) (see Barrett *et al*., 2005), where *σ*_G1_ and *σ*_G2_ are the standard deviations in the abilities of phage clones 1 and 2, respectively, to infect bacterial clones 1 and 2, and *ρ*_G1G2_ is environmental correlation across the two phage clones. Inconsistency was then averaged across structured populations and individual subpopulations. High inconsistency suggests that different phage clones are each adapted to infect a different subset of bacterial clones, indicating the evolution of host–parasite specialization. Responsiveness was calculated as *R *= *V*_PB_−*I*, where *V*_PB_ was calculated using a two‐way anova on clone performance. To test whether total diversity, and more importantly, host–parasite specialization differed across selection environments, we carried out one‐way anovas on the appropriate variance components. Using paired *t*‐tests, we then compared the degree of specialization in individual subpopulations vs. that of the population as a whole.

To meet model assumptions, response variables were transformed, where appropriate (see Results). In case of significant differences, we used Tukey contrasts to compare treatment means, with *α *< 0.05. We implemented the statistical package r Version 2.2.2 for all analyses (R Development Core Team; http://www.r-project.org).

### Mathematical models

Our intuition is that the reduction in diversity we have seen in our experiment can be explained by generalist pathogens being more likely to be favoured when specialists are more distantly related. To examine whether this idea is plausible, we built a simple model to examine, with as few assumptions as possible, the conditions under which a generalist is able to invade two specialist parasite strains that differ in how diverse they are. The aim of the model was to examine this scenario as an alternative outcome to the more general prediction of the converse that two specialist strains invade a generalist strain. Our aim was not a full analysis of the relative likelihood of different outcomes, but rather to determine whether a simple mechanism is a plausible explanation of our experimental findings. We therefore started by developing a coevolutionary model by assuming that two co‐existing host (bacteria) subpopulations, *S*_1_ and *S*_2_, coevolve with two specialist parasites (phage) with infected bacteria populations, *I*_1_ and *I*_2_. This captures the scenario we observed in the experimental set‐up where propagation in different media results in parasites having greater infectivity of hosts evolved in the same media. We initially assumed a high specificity between individual host and parasite strains similarly to a ‘matching‐alleles’ type infection function, where transmission is maximized when some parasite infection trait, *p*, exactly matches some host susceptibility trait, *h* (e.g. fig. 1 in Agrawal & Lively, 2002). There was then a Gaussian function around this maximum, meaning parasites show reduced transmission against ‘nearby’ hosts (see Appendix S1). We assumed this specialist continuous functional form since, although we were constrained to use a binary measure of infectivity in our coevolution experiments, we have observed substantial variation in phage infectivity across different host strains (i.e. phages can strongly or weakly inhibit growth in individual assays). As such, although we assumed that there is strong specificity, there is the potential for some infection of nearby strains.

We assumed that the population dynamics of the system are governed by a classic SI epidemiological model (see Appendix S1) and considered the coevolution of the hosts and their specialist parasites under the framework of adaptive dynamics (Dieckmann & Law, 1996; Marrow *et al*., 1996; Geritz *et al*., 1998; see also Best *et al*., 2009, 2010). As such, we assumed that small, rare mutations arise and attempt to invade the resident equilibrium (see Appendix S1). We examined whether a generalist parasite strain is able to invade and replace the two specialists at different points in the coevolutionary process using (i) a simplified model that does not account for costs to evolution, and (ii) a more general model that does include costs. We assumed that more generalism comes at a cost of lower infection than that achieved by a very specialist parasite. As such, we tested whether more divergence in the host is likely to select for more specialist parasites and therefore more diversity.

## Results

### Host–parasite specialization in the selection experiment

**Figure 2:**
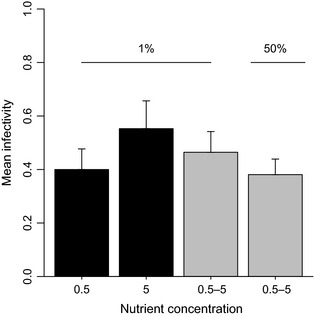
Mean (± SE) phage infectivity in relation to differential dispersal in homogenous (black bars) and heterogeneous (grey bars) selection environments.

**Figure 3:**
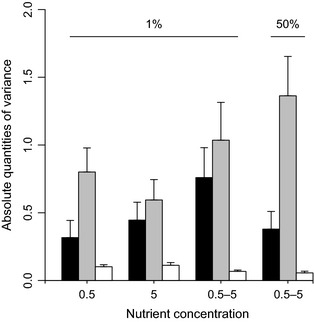
Mean (± SE) quantity of phenotypic variance attributable to effects of *V*_P_ (black bars), *V*_B_ (grey bars) and *V*_PB_ (white bars) across four different selection regimes.

**Figure 4:**
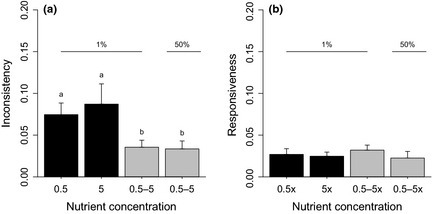
Mean (± SE) inconsistency (a) and responsiveness (b) in host–parasite populations coevolving in homogenous (black bars) and heterogeneous (grey bars) structured populations linked by dispersal. Letters denote significant differences (*P *<* *0.01).

Mean phage infectivity did not differ across treatments (one‐way anova:* F*_3,34_ **= 0.89, *P *=* *0.46; mean infectivity ± SE: *a* = 0.40 ± 0.08 (*n *=* *11), *b* = 0.55 ± 0.10 (*n *=* *7), *c* = 0.46 ± 0.08 (*n *=* *9), *d* = 0.38 ± 0.06 (*n *=* *11); Fig. 2). Although variation in mean infectivity among phage clones (*V*_P_) as well as variation of average phage infectivity across different bacterial clones (*V*_B_) did not differ across selection regimes (one‐way anova on √*x* data: *F*_3,34_ **= 1.25, *P *=* *0.31 and *F*_3,34_ **= 1.36, *P *=* *0.27 for *V*_P_ and *V*_B_, respectively; Fig. 3), phenotypic variance attributable to clone‐by‐clone interactions did (one‐way anova:* F*_3,34_ **= 3.39, *P *=* *0.03; Fig. 3). Crucially, the clone‐by‐clone variance attributable to inconsistency, a measure of within‐population host–parasite specialization, also varied significantly across selection regimes, being lower in heterogeneous environments (one‐way anova:* F*_3,34_ **= 3.71, *P *=* *0.02), and not differing between migration regimes (mean inconsistency ± SE: 0.075 ± 0.014, 0.084 ± 0.030 for the two homogeneous environments; and 0.036 ± 0.008 and 0.034 ± 0.009 for the two different migration treatments in the heterogeneous environment; Fig. 4a). A similar trend was found within individual subpopulations (one‐way anova:* F*_3,34_ **= 5.00, *P *=* *0.006; mean inconsistency ± SE: 0.037 ± 0.011, 0.048 ± 0.015 for homogeneous treatments and 0.010 ± 0.004, 0.006 ± 0.004 for heterogeneous treatments), but average inconsistencies were lower than that observed in the entire population (paired *t*‐test: *t *=* *5.12, *P *<* *0.0001), independent of the selection regime (one‐way anova on *I* differences: *F*_3,34_ **= 0.27, *P *=* *0.85). The proportion of total variance attributable to responsiveness did not vary across treatments (one‐way anova:* F*_3,34_ **= 0.34, *P *=* *0.80; Fig. 4b). In summary, phenotypic diversity in terms of resistance and infectivity range (number of parasites resisted or hosts infected) did not differ between treatments, but host–parasite specialization (presence of different phenotypes each specialized to infect/resist different hosts/parasites) was reduced in heterogeneous compared with homogeneous selection environments, regardless of migration rates.

### Mathematical models

**Figure 5:**
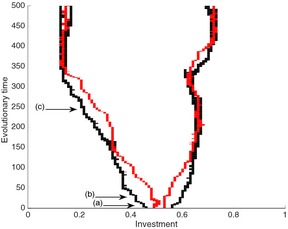
Example coevolutionary trajectory of hosts (black) and specialist parasites (red). The points marked (a–c) correspond to the values of *h* used in the plots in Fig. 6. Parameter values: *b = 2*,* q = 0.1*,* d = 1*,* α* = 1, *β*_S_ = 1, *ω *= 0.1. The output was generated by numerically solving the population dynamics (A.2)–(A.5) for a ‘resident’ strain using a Runge‐Kutta routine in the C programming language. These dynamics are run for large time such that it is approaching its dynamic attractor before an additional mutant strain is introduced at low density, and the dynamics are run again. At the end of each run, if any strain has fallen below a low threshold, it is assumed to be extinct.

An example coevolutionary trajectory of the two hosts and their specialist parasites is shown in Fig. 5 (see figure caption for details). Here, the two parasite strains begin ‘between’ the two host strains, which causes the hosts to diverge with one strain heading to ever lower values of trait *h* and the other to ever higher values. Similarly, the two parasite strains each ‘chase after’ one of the two host strains, becoming increasingly specialized. This relates to the experimental set‐up where coevolution in different media leads to divergence such that phages are much better at infecting bacteria grown in the same compared to different media (we note that spatial structure was neither required nor imposed for this result). We now consider the potential invasion of a generalist parasite in to this system, initially taking our simplified model with no costs. If a generalist parasite is to invade, it must have a positive growth rate whilst rare. We denote *β*_G_ and *β*_S_ as the generalist's and specialists’ respective maximal transmission rates, and *σ* the ‘overlap’ of the infection function (i.e. the reduction in transmission of a specialist parasite against a distant host). We can show (see Appendix S1) that in a model without costs that for this to be the case:

$$\beta_{G}>\frac{\beta_{S}\left(1+\sigma \right)}{2}.$$

The key element is that the transmission rate, *β*_G_, required by the generalist to invade increases with an increasing overlap of strains, *σ*. In other words, the generalist must achieve a combined transmission rate that is greater than the average of the specialists' transmission rates against the two hosts. Therefore, the generalist parasite could invade and replace two specialists provided the cost of generalism is not too large. We can also show that this simple and general result will hold when there are costs to investment, such that the two specialists may vary in their maximum transmission rate, *β*_S_ (but assume the shapes of the infection distributions remain identical), and that there is an associated cost to the parasite via a standard transmission–virulence trade‐off. This time it can be shown (see Appendix S1) that

$$\beta_{G}>\left(1+\sigma \right)\frac{\alpha_{G}+d}{X_{1}+X_{2}},$$

where *α*_G_ and *d* are the respective disease‐induced and natural mortality, and *X*_1_ and *X*_2_ are proportio‐nal to the susceptible densities (see Appendix S1). The key element here is that, again, the transmission rate, *β*_G_, required by the generalist to invade increases with an increasing overlap in infectivity ranges between strains, *σ*.

**Figure 6:**
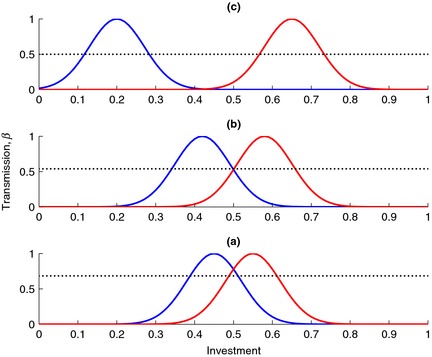
Example output from the simple model without costs showing the invasion potential of a generalist parasite in a system with two specialist parasites (blue and red lines) for different values of *h*_1_, *h*_2_ taken from the marked points in Fig. 5, and the minimum transmission rate required by the generalist to invade (dashed line). (a) *h*_1_ = 0.45, *h*_2_ = 0.55, (b) *h*_1_ = 0.42, *h*_2_ = 0.58, (c) *h*_1_ = 0.2, *h*_2_ = 0.65. Also, *β*_S_ = 1, *ω *= 0.1 in all plots.

We demonstrate the results from the simplified model graphically in Fig. 6, where three different points in the coevolutionary trajectory (see Fig. 5) are highlighted. Specialist parasites maximize their transmission at *β *= *β*_S_ when they ‘match’ their hosts, with reducing transmission against more dissimilar hosts (Fig. 6). Generalists must achieve greater transmission against each host than the average of the viral specialists against their specialist and nonspecialist hosts (i.e. if the specialist achieves transmission *σβ*_S_ against its nonspecialist host, the generalist must have transmission *β*_G_ > *β*_S_ (1 + *σ*)/2). Where the two hosts are very close (a), the specialist's distributions still overlap somewhat, meaning that the generalist parasite must achieve a transmission rate *β*_G_ > 0.684 *β*_S_ (*σ *= 0.368). As the hosts begin to diverge, the two infection distributions begin to overlap less and less, and the generalist needs not achieve so high a transmission rate to successfully invade. In (b), *β*_G_ > 0.539 *β*_S_ (*σ *= 0.078), and in (c), there is no overlap, such that *β*_G_ > 0.5 *β*_S_ (*σ* = 0). This very general process provides a simple explanation for the reduced diversity that is seen in the parasite in the presence of more diverse host types: a generalist parasite may incur higher costs of generalism and still invade two specialists when the resident hosts/parasite types are more diverse.

## Discussion

In this study, we experimentally determined if host–parasite specialization is enhanced in spatially heterogeneous vs. homogeneous abiotic environments, in coevolving populations of bacteria and viruses. Previous work has shown that the different environments used in the current study impose divergent selection on coevolving bacteria–phage populations, resulting in phages having greater infectivity on their own hosts than hosts evolved in the alternative environment (Lopez‐Pascua *et al*., 2012). As a result, we hypothesized that communities consisting of two different nutrient environments connected via migration would support more host–parasite specialization than populations where the nutrient environment is constant across subpopulations. However, we found the opposite to be true. Specialization was low in all populations, with most of the variation between bacteria and phage isolates explained by different mean resistance and infectivity ranges, respectively. We found that selection in heterogeneous environments, where dispersal links subpopulations following relatively divergent evolutionary trajectories, resulted in lower host–parasite specialization.

These results could not be explained by dispersal simply homogenizing populations. Such homogenization of populations can arise as a result of gene flow regardless of the mechanism responsible for local adaptation (i.e. genetic trade‐offs, accumulation of deleterious mutations in the nonselective environment or simply greater rates of adaptation to the environment in which genotypes spent the most time) (Kawecki, 1998; Lenormand, 2002). However, homogenization would have resulted in the heterogeneous treatments having levels of specialization equal to one of the homogeneous environments, not less than both of the homogenous environments.

We therefore developed a simple model to help explain these results. This model provides some key insights: generalist parasites were favoured precisely because the environments imposed divergent selection on hosts. This prediction is based upon two key assumptions. First, it assumes a cost to generalism, which is frequently observed in host–parasite systems (including this one), as a result of ecological or genetic trade‐offs (e.g. Turner & Elena, 2000; Thrall & Burdon, 2003; Poullain *et al*., 2008; Legros & Koella, 2010; Leggett *et al*., 2013). Second, it assumes that the specialism is not absolute, such that hosts that are similar to the host a parasite is specializing on can also be infected to some extent (Engelstadter & Hurst, 2006; Russell *et al*., 2009; Longdon *et al*., 2011). Taken together, these assumptions imply a nonlinear, step‐like relationship between host range and costs. As a result, it is only a net benefit to evolve costly generalism when hosts are sufficiently different, otherwise the specialist strategy will suffice (Legros & Koella, 2010). More generally, this can be explained by selection maximizing geometric mean fitness across heterogeneous environments to which all organisms are exposed (Via & Lande, 1985). Note that recent work (Guyader & Burch, 2008; Heineman *et al*., 2008; Sieber & Gudelj, 2014) has also emphasized the importance of optimal foraging theory (Pyke *et al*., 1977) for the evolution of generalists and specialists in the absence of any specific infectivity trade‐offs between hosts. Specifically, generalists may be selected against if some hosts result in lower yields of viruses, but may be selected for when under low host densities, in which case being able to infect as many hosts as possible is the optimal strategy. However, it is unclear how this could explain our results as host densities do not differ between environments and there is no evidence in this system that once infected different bacterial genotypes produce different yields of viruses (Benmayor *et al*., 2009).

We were initially surprised that the rate of gene flow had no significant effect on the degree of host–parasite specialization: greater generalism might be to evolve in populations that experience more intense gene flow (Lenormand, 2002) because of increasing homogenization of populations. However, despite migration rates being very different (50% and 1%), it is important to emphasize that these migration rates are imposed every approximately 13 generations, over which time major evolutionary changes occur: a resistant mutant can readily increase from undetectable levels to fixation over this period (Buckling & Rainey, 2002). In other words, even very high rates of migration only homogenize populations in the short term, after which populations rapidly diverge. Previous studies on coevolving *P. fluorescens* and *ϕ*2 have also demonstrated that low levels of gene flow are sufficient to maintain divergence between populations (e.g. Morgan *et al*., 2005).

In summary, we found that spatial heterogeneity reduced specialization, which is in contrast to the generally accepted paradigm. Previous work suggests that selection for differential infectivity/resistance strategies could play a key role in governing ecological interactions among populations, thereby affecting community structure and its responsiveness to environmental change (Bohannan & Lenski, 2000), the evolution of virulence (Leggett *et al*., 2013) and patterns of local adaptation (Gandon & Michalakis, 2002), all of which have important implications for human health and agriculture. Our finding of reduced diversity in heterogeneous environments may hold for any host–parasite system in which costs to parasite generalism show a step‐wise relationship with host range. Unfortunately, we are not aware of any studies where data are sufficiently extensive to unambiguously identify such relationships. Finally, it remains unclear whether our results apply to other trophic interactions, or evolution of single species, but if costs of generalism are also step‐like, they might.

## Supporting information


**Appendix S1** Mathematical modeling.
