# Supplementary material for: Spatial heterogeneity lowers rather than increases host–parasite specialization
Source: J Evol Biol. 2015 Jul 22;28(9):1682–90. doi: 10.1111/jeb.12689 (PMC4973826; doi:10.1111/jeb.12689)
Supplement: Supplementary file 1 — Appendix S1 Mathematical modeling. [file JEB-28-1682-s001.doc]

**Appendix S1: Mathematical modeling**

As stated in the main text, we wished to develop a simple mathematical model to explore how generalist parasites might invade a system of two specialists, and how the conditions for invasion might depend on how diverse the two existing specialists are. In this appendix we give full details of the modeling (note that parts of this text are repeated from the main text for clarity).

We developed a coevolutionary model by assuming that two co-existing host (bacteria) sub-populations, $S_{1}$ and $S_{2}$, coevolve with two specialist parasites (phage) with infected bacteria populations, $I_{1}$ and $I_{2}$. This captures the scenario we observe in the experimental set up where propagation in different media results in parasites having greater infectivity of hosts evolved in the same media. We initially assume a high specificity between individual host and parasite strains similarly to a `matching-alleles' type infection function, where transmission is maximized when some parasite infection trait, *p*, exactly matches some host susceptibility trait, *h* (e.g., Fig. 1 in Agrawal and Lively 2002). There is then a Gaussian function around this maximum meaning parasites show reduced transmission against ‘nearby’ hosts,

$$(A.1)\beta(h,p)=\beta_{S}\exp\left[-\left(\frac{p-h}{\omega}\right)^{2}\right]$$

where the maximum transmission rate of a specialist parasite is $\beta_{S}$ (see functions in Fig. 5 in the main text). We assume this specialist continuous functional form since, although we were constrained to use a binary measure of infectivity in our coevolution experiments, we have observed substantial variation in phage infectivity across different host strains (i.e., phages can strongly or weakly inhibit growth in individual assays). As such, although we assume that there is strong specificity there is the potential for some infection of nearby strains.

We assumed that the population dynamics of the system are governed by a classic SI epidemiological model as given by the following ordinary differential equations,

$$\begin{matrix} (A.2)\frac{dS_{1}}{dt}=(b-qN)S_{1}-\left[\beta(h_{1},p_{1})I_{1}+\beta(h_{1},p_{2})I_{2}\right]S_{1}-dS_{1} \\ (A.3)\frac{dS_{2}}{dt}=(b-qN)S_{2}-\left[\beta(h_{2},p_{1})I_{1}+\beta(h_{2},p_{2})I_{2}\right]S_{2}-dS_{2} \\ (A.4)\frac{dI_{1}}{dt}=\left[\beta(h_{1},p_{1})S_{1}+\beta(h_{2},p_{1})S_{2}\right]I_{1}-(\alpha+d)I_{1} \\ (A.5)\frac{dI_{2}}{dt}=\left[\beta(h_{1},p_{2})S_{1}+\beta(h_{2},p_{2})S_{2}\right]I_{2}-(\alpha+d)I_{2} \end{matrix}$$

All hosts are born susceptible at rate *b*, which is reduced due to crowding by the density dependent term *qN* where *N* is the total population density. All hosts die at rate *d*, with infected hosts suffering additional mortality (virulence) at rate $\alpha$. We assumed no recovery and no reproduction by infected hosts. Transmission is a mass action process between susceptible and infected hosts with coefficient $\beta(h,p)$ as given in equation (A.1). In this initial model, we have assumed that the two specialist parasite strains have identical infection distributions, with the same value of $\beta_{S}$ against their specialist hosts (when *h=p*).

We considered the coevolution of the hosts and their specialist parasites under the framework of adaptive dynamics (Dieckmann and Law 1996; Marrow et al. 1996; Geritz et al. 1998; see also Best et al. 2009; Best et al. 2010). As such, we assumed that small, rare mutations arise and attempt to invade the resident equilibrium. Considering the growth rates of such mutants the fitnesses of the mutant host and parasite strains are respectively given by,

The coevolutionary trajectory of this system is determined by the relative mutation rate of each strain multiplied by the local selection gradients,

Given the definition of $\beta(h,p)$ in equation (A.1) it is easy to show that we have,

The interpretation of the selection gradients (A.10) and (A.11) is that the host will always try to evolve away from the parasite (weighted by whichever parasite strain is ‘nearer’) and the parasite will always try to evolve towards the host.

On top of this underlying coevolutionary model, we then examined whether a generalist parasite strain is able to invade and replace the two specialists at different points in the coevolutionary process using (1) a simplified model that does not account for costs to evolution, and (2) a more general model, which does include costs. We assume that more generalism comes at a cost of lower infection than that achieved by a very specialist parasite. As such we tested whether more divergence in the host is likely to select for more specialist parasites and therefore more diversity.

An example coevolutionary trajectory of the two hosts and their specialist parasites is shown in Fig. 5 in the main text (see figure caption for details of the simulation process). Here, the two parasite strains begin ‘between’ the two host strains, which causes the hosts to diverge with one strain heading to ever lower values of trait *h* and the other to ever higher values. Similarly, the two parasite strains each ‘chase after’ one of the two host strains, becoming increasingly specialized. We show an additional figure (A.1) here that highlights the region of coexistence for the two specialist parasites at a snapshot of evolutionary time (point (b) from Figs. 5 and 6 in the main text), highlighting the wide potential for such a situation to arise.

Figure A.1 – The region of coexistence for the two specialist parasite types at a snapshot of evolutionary time (black regions). Parameters are as of Fig. 5 in the main text, with *h1=0.42, h2=0.58* marked by the white dots (i.e. point (b) from Figs. 5 and 6).

We now consider the potential invasion of a generalist parasite in to this system, initially taking our simplified model with no costs. If a generalist parasite is to invade, it must have a positive growth rate whilst rare, that is,

$$(A.12)r=\beta_{G}(S_{1}+S_{2})-(\alpha+d)>0.$$

Considering equations (A.4) and (A.5) and assuming that the specialist parasites are currently maximizing their transmission on their respective hosts and have reduced transmission against the second host by an amount $0<\sigma<1$ (as given by equation (A.1)), it is simple to show that at equilibrium,

$$(A.13)S^{*}=S_{1}=S_{2}=\frac{\alpha+d}{\beta_{S}(1+\sigma)}$$

meaning that (A.12) becomes,

$$\begin{matrix} (A.14)r=2\beta_{G}S^{*}-\beta_{S}(1+\sigma)S^{*}>0 \\ (A.15)\Rightarrow\beta_{G}>\frac{\beta_{S}(1+\sigma)}{2} \end{matrix}$$

In other words, the generalist must achieve a combined transmission rate that is greater than the average of the specialists' transmission rates against the two hosts. Therefore the generalist parasite could invade and replace two specialists provided the cost of generalism is not too large. Furthermore, as the two specialists become more diverse, and therefore $\sigma$ becomes smaller, the generalist is able to incur higher costs and still invade.

We can also show that this simple and general result will hold when there are costs to investment, such that the two specialists may vary in their maximum transmission rate, $\beta_{S}$ (but assume the shapes of the infection distributions remain identical), and that there is an associated cost to the parasite via a standard transmission-virulence trade-off. In this case, the growth rate is given by,

$$(A.16)r=\beta_{G}(S_{1}+S_{2})-(\alpha_{G}+d)>0.$$

and by considering the revised equations (A.4) and (A.5) we have,

$$(A.17)(S_{1}+S_{2})(1+\sigma)=\frac{\alpha_{1}+d}{\beta_{S_{1}}}+\frac{\alpha_{2}+d}{\beta_{S_{2}}}=X_{1}+X_{2}$$

The fitness then does not reduce to as simple an expression as previously, but we can write,

$$\begin{matrix} (A.18)r=\frac{\beta_{G}}{1+\sigma}(X_{1}+X_{2})-(\alpha_{G}+d)>0 \\ (A.19)\Rightarrow\beta_{G}>(1+\sigma)\frac{\alpha_{G}+d}{X_{1}+X_{2}} \end{matrix}$$

where and *d* are the respective disease-induced and natural mortality, and and are proportional to the susceptible densities. The key element here is that, again, the transmission rate, $\beta_{G}$, required by the generalist to invade increases with an increasing overlap of strains, $\sigma$.

We demonstrate the results from the simplified model graphically in Fig. 6 in the main text. This figure shows how the generalist can invade with a lower transmission rate (that is, a higher cost to generalism) as the two specialists become more diverse. We show an additional figure (A.2) here demonstrating how the transmission rate required by the generalist depends on the difference in trait value between the two specialist hosts/parasites (c.f. eqn (A.15)). Clearly, as the two specialists become more diverse, the generalist parasite can invade with a lower transmission rate (i.e. higher costs of generalism).

Figure A.2 – The level of transmission, $\beta_{G}^{*}$, required by a generalist parasite to invade a system as the difference between the two specialists varies. Parameters: $\beta_{S}=1,\omega=0.1$.
